# Early transfusion strategies in haemorrhagic shock

**DOI:** 10.1186/s13049-026-01568-7

**Published:** 2026-02-23

**Authors:** Ingrid Nygren Rognes, Marius Rehn

**Affiliations:** 1 Department of Anaesthesiology and Intensive Care Medicine, Akershus University Hospital, Lørenskog, Norway; 2 Department of Research and Development, Norwegian Air Ambulance Foundation, Oslo, Norway; 3 Institute of Clinical Medicine, University of Oslo, Oslo, Norway; 4 Division of Prehospital Services, Oslo University Hospital, Oslo, Norway

## Main text

Major haemorrhage is a leading cause of morbidity and mortality in various medical settings including trauma, vascular emergencies, gastrointestinal bleeding and obstetric complications. Crucial aspects of optimising the pre- and early in-hospital management of these patients remain scientifically unresolved. In this collection of articles, the Scandinavian Journal of Trauma, Resuscitation and Emergency Medicine presents a series of studies that aim to enhance our understanding of early transfusion strategies in haemorrhagic shock.

### Identification and scoring systems

Pivotal for managing major haemorrhage is the identification of patients in need of urgent and massive transfusion. Early clinical evaluation remain challenging and vital signs may be paradoxical to physiologic state, making them unreliable to predict volume of bleeding, need for transfusion and outcome [1]. «Clinical gestalt» has also shown both low sensitivity and specificity to predict the need for massive transfusion [2].

Various scoring systems are being used by clinicians and researchers to predict the need for massive transfusion, major interventions and prognosis in bleeding trauma patients. Tran et al. evaluated the performance of the Assessment of Blood Consumption (ABC), Trauma Associated Severe Hemorrhage (TASH) and Shock Index (SI) scores across a range of bleeding-related outcomes. They found that all three performed inadequately in the discrimination of massive transfusion, the need for haemostatic interventions and haemorrhage-related mortality, confirming well-established limitations of the existing bleeding scores and highlighting the need for improved tools [3].

Laboratory and radiological examinations are easily accessible in most hospital systems and may, together with clinical assessment, support decision making. Tran et al. presented a refined version of the Canadian Bleeding (CAN-BLEED) score [4] that combines vital signs, laboratory measures, clinical examination and radiological findings, to predict major interventions in trauma patients. This refined scoring system showed excellent performance in identifying patients requiring major interventions for traumatic bleeding [5]. Viscoelastic haemostatic assays are variously being used to support decision making in massive transfusion protocols. However, Coggins et al. found that point of care viscoelastic haemostatic assays alone were not sufficient to reliably predict massive transfusion in a small cohort of bleeding trauma patients in an Australian emergency department setting [6].

Jänig et al. validated the Reverse Shock Index multiplied by Glasgow Coma Scale (rSIG) and ABC scores in a pre-hospital setting utilising retrospective German TraumaRegister DGU® (TR-DGU®) data and found both scoring systems useful in predicting massive pre-hospital transfusion in trauma patients. Moreover, they developed the pre-hospital Trauma Associated Severe Hemorrhage (ph-TASH) score with variables from rSIG and ABC-scores, that showed even better performance [7]. Pre-hospital Focused Assessment with Sonography in Trauma (FAST) examination was seemingly the most decisive for prediction in their analyses. The ph-TASH score will be implemented in several German Helicopter Emergency Medicine System (HEMS) stations, arguing for HEMS crews to implement ultrasound and FAST examinations in routine practice.

Identification of paediatric patients in need of transfusion can be harder and distinctive predictors for the need of transfusion in paediatric trauma in a pre- and early in-hospital setting are lacking. Schneider et al. found in multivariate regression analysis on German TR-DGU® data from 11 000 children and adolescents that massive transfusion was extremely rare, and that polytrauma, abdominal trauma and penetrating trauma were predictors for erythrocyte concentrate transfusion [8]. Glasgow Coma Scale ≤ 8, and the need for endotracheal intubation or cardiopulmonary resuscitation were also significant, although weaker, predictors.

### Transfusion products and aetiology

Alongside haemorrhage control, maintenance of an adequate intravascular blood volume is the mainstay of initial therapy for patients in haemorrhagic shock. Transfusion strategies have changed over the last few decades from erythrocyte concentrates and crystalloids to balanced transfusion with erythrocyte concentrates, plasma and thrombocyte concentrates or whole blood [9]. The PROPPR [10] and PAMPer [11] trials have shown benefits of early, pre-hospital plasma transfusion, whereas the RePhill trial [12] later failed to show benefit for erythrocyte and lyophilised plasma over crystalloid infusion. Mitra et al. showed that among trauma patients receiving massive transfusion, high ratios of plasma to erythrocyte concentrates were associated with lower mortality in a secondary analysis of the PATCH-trauma data [13]. Non-traumatic bleeding is underrepresented in literature, especially in the pre-hospital setting. Kodakadath et al. retrospectively described a small cohort of non-traumatic bleeding patients from the HEMS base in Sussex, England, and found that pre-hospital blood transfusion was most common in patients with gastrointestinal bleeding, followed by unidentified bleeding sources and postpartum haemorrhage [14].

### Transfusion training

Safe and effective pre-hospital blood transfusion is a pivotal initiative that may also be performed by non-physician personnel. In a scoping review Dion et al. summarised literature on blood transfusion training and found that training is also feasible for non-physician pre-hospital providers, delivering improved knowledge, skills and confidence [15]. Long-term retention and effects on clinical outcome are however not well elucidated in current literature. By surveying the national Critical Care Transport Organizations in Canada, the same group found that existing pre-hospital transfusion training varies, particularly regarding competence assessment, certification renewal and patient outcome, and now aim to establish national training standards [16]. Given the current global variability in pre hospital staffing, trained non-physician personnel may be required to provide blood transfusions in austere settings and further training is warranted in both military and civilian settings.

### Hypocalcemia

Hypocalcemia is prevalent and may prove critical in trauma patients with major haemorrhage. Calcium supplementation to avoid hypocalcemia during trauma resuscitation remains integrated in transfusion guidelines. An adequate level of serum ionised calcium is crucial for the coagulation cascade and platelet function, and moreover for normal regulation of vasomotor tone and cardiac contractility. Srichuachom et al. here presented an updated systematic review and meta-analysis where hypocalcemia on emergency department arrival affected approximately half of all adult major trauma patients and was associated with increased mortality [17]. The effect of calcium supplementation during trauma resuscitation has earlier failed to show an effect on early mortality and also hypercalcemia has been associated with worse outcome [18]. Hibberd et al. performed a cross-sectional survey to explore the existence and content of standard operating procedures (SOPs) for measurement and treatment of hypocalcemia during pre-hospital blood transfusion in UK HEMS. 20 out of 21 HEMS had an SOP for calcium replacement during pre-hospital blood transfusion, whereas the exact guidance on when to replace was missing in a quarter of them. Only 6 out of 21 HEMS had a point of care test (POCT) available [19]. Pre-hospital POCT have several limitations as it may be time consuming and factors such as temperature and vibration, as well as sampling and machine challenges, may interfere. Empiric use of transfusion supplemental pharmaceuticals can be necessary but, as seen in the CRYOSTAT-2 study, empiric use of fibrinogen was not beneficial for all patients [20]. This may in theory also be the case for calcium supplementation, and perhaps is monitoring valuable.

### Conclusion

Transfusion of blood products has become mainstay in resuscitation of patients with major haemorrhage also in the pre-hospital setting. There is a paucity of data from patients with haemorrhage from aetiologies other than trauma and this limitation remains carried through this collection of articles. Identification of whom to transfuse and when to transfuse is challenging. Ultrasound examination may be useful in the pre-hospital and early in-hospital phase to identify patients in need of urgent transfusion and major interventions, in combination with clinical evaluation and scoring systems. Further ultrasound competence is called for. An increased awareness of calcium levels in trauma resuscitation is warranted and POCT may be necessary to avoid the unknown consequences of empiric use.

## Data Availability

No datasets were generated or analysed during the current study.

## Abbreviations

ABC: Assessment of Blood Consumption
TASH: Trauma Associated Severe Haemorrhage
SI: Shock Index
CAN-BLEED: Canadian Bleeding
rSIG: Reverse Shock Index multiplied by Glasgow Coma Scale
TR-DGU®: TraumaRegister DGU®
ph-TASH: Pre-hospital Trauma Associated Severe Hemorrhage
FAST: Focused Assessment with Sonography in Trauma
HEMS: Helicopter Emergency Medicine System
SOPs: Standard Operating Procedures
POCT: Point of Care Test
